# Effectiveness of mobile-phone short message service (SMS) reminders for ophthalmology outpatient appointments: Observational study

**DOI:** 10.1186/1471-2415-8-9

**Published:** 2008-05-31

**Authors:** Elizabeth Koshy, Josip Car, Azeem Majeed

**Affiliations:** 1 Department of Primary Care and Social Medicine, Imperial College London, London, UK

## Abstract

**Background:**

Non-attendance for hospital outpatient appointments is a significant problem in many countries. It causes suboptimal use of clinical and administrative staff and financial losses, as well as longer waiting times. The use of Short Message Service (SMS) appointment reminders potentially offers a cost-effective and time-efficient strategy to decrease non-attendance and so improve the efficiency of outpatient healthcare delivery.

**Methods:**

An SMS text message was sent to patients with scheduled appointments between April and September 2006 in a hospital ophthalmology department in London, reminding them of their appointments. This group acted as the intervention group. Controls were patients with scheduled ophthalmology appointments who did not receive an SMS or any alternative reminder.

**Results:**

During the period of the study, 11.2% (50/447) of patients who received an SMS appointment reminder were non-attenders, compared to 18.1% (1720/9512) who did not receive an SMS reminder. Non-attendance rates were 38% lower in patients who received an SMS reminder than in patients who did not receive a reminder (RR of non-attendance = 0.62; 95% CI = 0.48 – 0.80).

**Conclusion:**

The use of SMS reminders for ophthalmology outpatient appointments was associated with a reduction of 38% in the likelihood of patients not attending their appointments, compared to no appointment reminder. The use of SMS reminders may also be more cost-effective than traditional appointment reminders and require less labour. These findings should be confirmed with a more rigorous study design before a wider roll-out.

## Background

Non-attendance for hospital outpatient appointments is a major burden on healthcare systems and costs the National Health Service (NHS) in the UK an estimated £790 million per year [1]. It reduces the efficiency and effectiveness of the delivery of outpatient healthcare and causes substantial financial losses for healthcare systems [2]. It also results in suboptimal use of clinical and administrative staff and results in increased waiting times for other patients [3]. The increased waiting time can result in delay in presentation of patients' symptoms and also decreased monitoring of long-term chronic conditions; which can, in turn, lead to increased patient morbidity [4]. Additionally, there are worse outcomes for non-attenders and a loss of continuity of care [5].

Some of the main reasons for patients not attending their outpatient appointments are forgetting their appointments [6] and confusion over the date, time and location of the appointment [6,7] There are many methods of delivering appointment reminders which have been studied to date, including personalised and automated telephone reminders [6,8-12], posted letters [13,9] and awareness campaigns [14]. Most of the studies have shown a reduction in non-attendance rates, irrespective of the method of reminder used. However, there is a paucity of research in relation to the use of mobile phone SMS (Short Message Service)/text message reminders for outpatient hospital appointments [3].

As mobile phone ownership continues to increase rapidly in many countries worldwide, there is potential to utilise SMS reminders to increase the effectiveness and efficiency of health care delivery. A recent survey showed that over 85% of adults in the UK used a mobile phone [15] SMS facilities on mobile phones were introduced in the early 1990s and now represents one of the most widely used methods of communication, with about 41.8 billion texts sent in the UK in 2006 [16]. SMS messages have a number of characteristics that make them very appropriate for use in a healthcare setting including: direct patient communication, privacy, confidentiality, swift delivery of messages and receipt of responses, convenience for health providers and patients. SMS messaging technology also allows the dispatching of substantial numbers of messages simultaneously, so reducing labour expenditure.

Non-attendance at hospital outpatient clinics is a common problem which every specialty faces. It is particularly important for specialties such as ophthalmology which are predominantly outpatient-based [17] An audit in an ophthalmology clinic in a Birmingham hospital in the UK, reported a non-attendance rate of 12.6% [18]. A New Zealand study found non-attendance rates at a public eye clinic of 17.2% [17]. This highlights that it is essential to maximise patient attendance to optimise the efficiency of the service delivered. We could not identify any studies relating to the use of SMS-based reminders for hospital appointments for ophthalmology appointments within the Medline, Embase, Cochrane, and PsycInfo databases.

This pilot study primarily focused on patients attending ophthalmology clinics as these outpatient clinics had one of the highest non-attendance rates. Our study assessed the effectiveness of the use of SMS-based reminders for hospital outpatient appointments as a method of reducing the non-attendance rates in an inner-city London teaching hospital. The null hypothesis was that (non-)attendance rates for those receiving an SMS reminder did not differ from the (non-)attendance rates for those who did not receive an SMS reminder.

## Methods

This is an observational study and analysis is based on data collected from the ophthalmology department at Barts and the London Hospitals NHS Trust, UK between April and September 2006. Ethics committee approval was obtained from Barts and The London NHS Trust for SMS reminders to be sent to patients. We analysed data that did not contain any patient identifiable information.

All patients, where a mobile number was obtainable from the Patient Administration System and who were due to attend their first (new) ophthalmology consultation during the study period, were sent an automated SMS reminder. The SMS reminders were sent one day before the appointment if it was booked within seven days of the appointment. If an appointment had been booked more than seven days in advance of an appointment, the patient received an SMS four days beforehand. A four day interval was chosen to minimise the time delay for patients to still forget their appointments, but also to allow time to reschedule appointments, if patients subsequently cancelled their appointment. Information required for the SMS reminders was obtained from the Patient Administration System hospital database. This included the patients' mobile numbers, appointment dates and times. Patients who received an SMS formed the intervention group. A control group were all patients who had a scheduled first (new) appointment during this same study period, who may or may not have had a mobile phone, but whose mobile number was not available and did not receive any other form of appointment reminder.

The text message read: "*This is a reminder of your appointment at Barts and the London Hospital at <time> <date>. Please call xxxxx or reply to text to cancel*."

The automated SMS reminders transmitted were timed so that they did not reach the recipients at inappropriate times, such as night-time. The attendance/non-attendance status for patients who received and did not receive an SMS appointment reminder were recorded using the IT software installed. Data on cancellations by patients for the SMS and control groups was also collected.

Statistical analysis was performed using Stata 9. The attendance rates in the SMS reminder group were compared with those in the control group. A chi-squared test was conducted to compare the proportions of patients not attending appointments and also the proportions of cancellations by patients between the SMS and the control groups. Relative risks of non-attendance are presented with 95% confidence intervals (CI).

## Results

**Figure 1:**
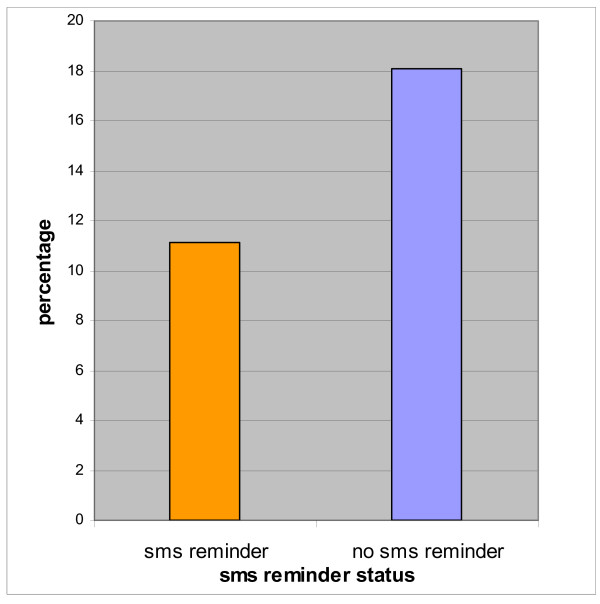
Percentage of non-attenders by SMS reminder status.

Of the 9959 ophthalmology hospital outpatient appointments between April and September 2006, 447 patients (4.5%) received an SMS appointment reminder (Table 1). The control group (patients who did not receive an SMS reminder) consisted of 9512 patients and had a non-attendance rate of 18.1% (1720/9512). Amongst the patients who received an SMS reminder (447 patients), the non-attendance rate was 11.2% (50/447) (Table 1, Fig 1). The absolute reduction in non-attendance rates for those who received an SMS reminder was 6.9%. This represents about 31 more (of the 447 ophthalmology outpatient appointments) being kept over a 6 month period that would have otherwise been lost through patients not keeping their scheduled appointments. The cancellation rates of appointments by patients who received an SMS and those who did not receive an SMS were 13.4% and 11.2%, respectively (Table 1).

**Table 1:** Non-attendance rates for patients sent a SMS reminder and those not sent a SMS reminder

**SMS appointment reminders**			**No SMS appointment reminders**			**Non-attendance rate reduction (%) with SMS reminders**
		
***TOTAL outpatient appointments***	***Non-attendance appointment numbers (%)***	***Cancellations by patients (%)***	**TOTAL outpatient appointments**	**Non-attendance appointment numbers (%)**	**Cancellations by patients (%)**	
447	50 (11.2)	60 (13.4)	9512	1720 (18.1)	1016 (10.7)	6.9

N = 9959

Patients who received a text reminder were 38% less likely to be non-attenders for their appointment (relative risk of non-attendance = 0.62; 95% CI 0.48 – 0.80, p = 0.0002). Patients in the SMS group were also 26% more likely to cancel their appointments, but this was not statistically significant (relative risk of cancellation 1.26, 95% confidence interval 0.98 – 1.61, P = 0.068). There was a total of 340,700 scheduled outpatient appointments (for all specialities) between April-September 2006. Of the 340,146 who did not receive an SMS appointment reminder the non-attendance rate was 12.1%.

The cost of sending an automated SMS reminder was 7.2 pence. Therefore, extrapolating the six month data to one year for the ophthalmology clinics, sending 894 SMS reminders (447 × 2) would have cost £64.37. The cost of approximately 62 (31 × 2) extra appointments being attended as a consequence of the SMS reminders, is £4030 (with a cost of a hospital appointment in England being approximately £65.00). Therefore, the net saving would have been £3965.63, just for the ophthalmology department. The 'number needed to text' (NNT) in this study to prevent one appointment non-attendance is 14 (95% confidence interval: 10 to 31).

## Discussion

Patients who had received an SMS reminder were significantly more likely to attend their ophthalmology hospital appointments compared to those who had not received an SMS. This study showed that sending SMS reminders led to a 6.9% absolute reduction and 38% relative reduction in non-attendance rates for ophthalmology outpatient appointments. Non-attendance rates in the SMS group and the control group were 11.2% and 18.1%, respectively. The cancellation of appointments in the SMS group compared to the control group was slightly higher (13.4% vs 11.2%). Cancellation rates were not statistically significantly different between the two groups but there was a trend towards a higher cancellation rate in the SMS group (relative risk of cancellation 1.26; 95% CI 0.98–1.61).

The non-attendance rate for ophthalmology appointments (18.1%) in our control group was higher than the mean non-attendance rate for all the different hospital specialty outpatient appointments (12.1%). This confirmed that non-attendance was a greater problem for the ophthalmology department compared to many other specialties within the same hospital. It is difficult to speculate why this might be, but there could have been administration problems, for example, due to staff shortages; or perhaps, though this may be unlikely, the demographic characteristics or behaviours of the patients in this geographical area who attend the ophthalmology clinics may differ from that of other specialties. The 18.1% non-attendance rate in the control group in our study is higher than that quoted in other studies [19,18,17]. For example, the Birmingham ophthalmology department audit, over a year, was 12.6% [18]. However, our study was only conducted over a 6 month period and ideally, information relating to a year, taking into account seasonal variations, would be valuable. Also, this Birmingham study was in 1990–1, so non-attendance rates may have changed over this time period. A New Zealand audit showed a non-attendance rate of 17.2% [17].

The non-attendance rate of the SMS group in our study was 38% lower than that of the control group (RR = 0.62) and there was strong evidence (P = 0.0002) to reject the null hypothesis of no difference between these two groups. A multi-centred, randomised controlled trial in China also found an increase in the likelihood of attendance (OR = 1.59, P = 0.005) [20]. This study had 993 participants who were divided into 3 study arms; and compared SMS reminders and mobile telephone conversations reminders with a control group of no intervention. There was no statistically significant difference in non-attendance observed between their SMS group and mobile phone-call reminders. This study was conducted in a primary care setting, which may have represented a systematically different group of patients in terms of non-attendance characteristics to those attending secondary care appointments, so direct comparisons cannot be made. Additionally, Family Practice-based factors such as mistakes or misunderstandings surrounding appointment details, relayed over the telephone, from the health provider are possible reasons for non-attendance in this healthcare setting [21].

The percentage reduction in non-attendance rates with SMS reminders of 6.9% in our study is lower than that reported for a recent study in an Irish ENT outpatient department [2], where there was a 11.6% reduction (from 33.6% to 22%). This study, however, used data from a much larger sample size (3981 patients) and covered a 3 year period. Studies of other methods of appointment reminders such as posted letters and telephone calls found reductions of non-attendance rates between 6% and 19% [6,8,12,10]. The reduction in non-attendance found in our study is within the range of these other studies. Therefore, SMS reminders are at least as effective as alternative methods. The SMS reminders also have the advantage of being more cost-effective and requiring less labour than the other methods.

### Limitations of this study

Although there seems to be an association between the use of SMS reminders and a decrease in non-attendance rates, this is not necessarily causal and there could be confounding factors and biases which may partially or fully explain this association. This study suggests that the use of SMS reminders could help to reduce ophthalmology non-attendance rates. However, the design in this paper is sub-optimal, and a rigorous randomised controlled trial, stratified by socio-demographic characteristics and clinical conditions, is required to determine if the potential benefits observed in this study can be replicated. Knowledge of the patients' clinical presentation in the intervention and control groups is also important as it could affect the 'value' placed on it by patients and potentially affect the likelihood of an appointment being kept.

We do not know what proportion of the control group owned mobile phones, but whose mobile number was not available for this service. We are also unaware of the number of patients who were unfamiliar with using text-messaging and so unable to read the reminder. There could also have been participation bias, as the SMS group patients (who provided a mobile phone number) may have been a more motivated group of patients; and these patients may have been more likely to attend their appointments irrespective of receiving an SMS reminder. This could have potentially led to an over-estimate of the association between SMS reminders and non-attendance rates. Data was only available for a 6 month period, and so did not take account of seasonal and monthly variations in non-attendance. However, using the same time-frame for the controls helped to reduce the chance of monthly and seasonal variations contributing to the observed differences. We did not have demographic information relating to those patients who owned a mobile phone or for those who received an SMS reminder who subsequently attended and did not attend their scheduled appointments. Data relating to potential confounding factors (such as age, sex, ethnicity and socio-economic status) was not collected, so we were not able to adjust for these in the statistical analysis. We did not have demographic information available relating to age in either groups and recognise this is a weakness and could account, in part or fully, for the observed difference. However, as younger patients are more likely to own mobile phones and also have higher non-attendance rates, the fact that the non-attendance rate decreased in the SMS group (who are more likely to be younger), suggests that use of SMS reminders may be beneficial to target younger patients. Finally, patients who received SMS reminders were for a first consultation and it has been suggested that follow-up appointments have higher non-attendance rates [22]. This was the first stage of the study; the next stage will be focusing on follow-up appointments.

### What this study adds

SMS appointment reminders seem to be an effective and efficient method of improving ophthalmology outpatient attendance, which is less labour intensive than the more traditional reminder systems that have been used in the past. The facility for patients to reply or call in response to an SMS reminder to cancel their appointment can help to reduce the non-attendance rates and free up appointments for other patients, which otherwise may be lost. We cannot extrapolate our findings to other specialties or hospitals; so future research should include suitably powered randomised controlled trials for assessing SMS appointment reminders for ophthalmology and other specialties to assess the cost-effectiveness within the NHS. The use of SMS technology is expanding and has already been used to help in the management of some health problems and diseases; for example, asthma [23] and diabetes [24,25] management and smoking cessation [26]. Hence, the potential scope for the further utilisation of this technology is tremendous. SMS reminders could offer a cost-effective method for reducing non-attendance rates for retinal screening appointment follow-ups for diabetic patients.

### Strengths and weaknesses of SMS technology

In addition to the advantages already described, SMS reminders require minimal investment in IT infrastructure, as this is already in existence; as the IT software for sending automated SMS integrates with existing electronic patient health records and hospital administrative database systems. Once the system is in place, the cost of running the service increases very little as the number of SMS reminders increases. As they are automated messages, it does not require staff training so this offers a time, labour and cost-efficient system.

There are a number of potential weaknesses. Patients may not receive the SMS reminders due to incorrect data entry. However, this problem can occur with other methods of appointment reminders such as letters and telephone calls (with change of address) [12]. The uses of different technologies are very fluid and dynamic and so it is not known how the use of mobile phones and the use of SMS technology will evolve over time. However, it is anticipated that there will be an increase in mobile phone ownership and use of SMS messaging facilities in the immediate few years. Elderly people have lower ownership rates of mobile phones and may not be able to use SMS facilities [27]. The Office for National Statistics (UK) [27] found mobile ownership in 2003 varied by age, with nearly 90% of 15–24 year olds owning one and less than a quarter of those aged 75 years and over owning one in 2003. However, between 2001 and 2003, the largest increases in ownership were amongst those aged 75 and over, with the proportions almost doubling [27]. However, it has traditionally been younger patients who have missed their outpatient appointments more than older patients, so the use of SMS reminders could be targeted towards patients under 60 years old.

### Cost-effectiveness analysis

There is always some wastage in any system and the real cost of a missed appointment is difficult to quantify. It may be, in some cases, that the appointment may not have really been needed, although this is difficult to prove. For example, patients' agenda may influence presentation and attendance [28]. Based on the 'number needed to text' analysis, approximately fourteen people would need to be sent an SMS reminder to prevent one non-attendance. As the cost of outpatient appointments is considerable, this could potentially be worthwhile, but needs to be tested through a more rigorous economic analysis. A well-designed randomised controlled trial would give a more accurate representation of the NNT to prevent one non-attendance. Only around 5% of the patients with scheduled ophthalmology appointments were sent SMS reminders in this study; so, if the reminders were sent on a much larger scale to patients with first and follow-up appointments and for all the departments in the hospital, the savings to the NHS could be large. A previous cost-effectiveness analysis has estimated that the annual direct cost of missed hospital appointments in England is estimated to be close to £575 million [1] and that the use of SMS-based reminders could lead to a potential saving of £55.6 to £83.5 million a year [1], so the potential scope for this technology is considerable.

## Conclusion

The use of SMS reminders for ophthalmology outpatient appointments was associated with a reduction of 38% in the likelihood of patients not attending their appointments, compared to no appointment reminder. Additionally, the use of SMS reminders appears more cost-effective than traditional appointment reminders and requires less labour. These findings suggest that SMS text reminders have great potential value in reducing non-attendance rates in outpatient departments but that the potential benefits should be confirmed with a more rigorous study design before a wider roll-out.

## Competing interests

The authors declare that they have no competing interests.

## Authors' contributions

EK and JC conceived the idea for this paper. EK wrote the manuscript. EK and AM performed the statistical analysis and data interpretation. JC and AM were involved in interpretation of findings and revised the paper critically for intellectual content. All authors read and approved the final manuscript. EK and JC are the guarantors for this study.

## Pre-publication history

The pre-publication history for this paper can be accessed here:


